# Reproductive toxicity of fluoroquinolones in birds

**DOI:** 10.1186/s12917-019-1957-y

**Published:** 2019-06-21

**Authors:** Hana Hruba, Ehdaa Eltayeb Eltigani Abdelsalam, Nikolay Anisimov, Hana Bandouchova, Barbora Havelkova, Tomas Heger, Miroslava Kanova, Veronika Kovacova, Monika Nemcova, Vladimir Piacek, Jana Sedlackova, Frantisek Vitula, Jiri Pikula

**Affiliations:** 1 Department of Ecology and Diseases of Game, Fish, and Bees, Faculty of Veterinary Hygiene and Ecology, University of Veterinary and Pharmaceutical Sciences Brno, Palackeho tr. 1946/1, 612 42, Brno, Czech Republic; 2 Institute of Environmental and Agricultural Biology (X-BIO), Tyumen State University, Volodarskogo 6, 625003 Tyumen, Russia; 3 CEITEC - Central European Institute of Technology, University of Veterinary and Pharmaceutical Sciences Brno, Brno, Czech Republic

**Keywords:** Antibiotics, Enrofloxacin, Marbofloxacin, Reproduction, Hatchability, Pre-term hatching, Avian embryonic heart rate

## Abstract

**Background:**

While commercial poultry and captive birds are exposed to antimicrobials through direct medication, environmental pollution may result in contamination of wild birds. Fluoroquinolones are commonly used medications to treat severe avian bacterial infections; however, their adverse effects on birds remain understudied. Here, we examine toxicity of enrofloxacin and marbofloxacin during the egg incubation period using the chicken (*Gallus Gallus domesticus*) as a model avian species. Laboratory tests were based on eggs injected with 1, 10 and 100 μg of fluoroquinolones per 1 g of egg weight prior to the start of incubation and monitoring of chick blood biochemistry, reproductive parameters and heart rate during incubation.

**Results:**

Eggs treated with fluoroquinolones displayed reduced hatchability due to embryonic mortality, particularly on day 13 of incubation. Total hatching success showed a similar pattern, with a significantly reduced hatchability in low and high exposure groups treated with both enrofloxacin and marbofloxacin. From 15 to 67% of chicks hatching in these groups exhibited joint deformities. Hatching one-day pre-term occurred with a prevalence of 31 to 70% in all groups treated with fluoroquinolones. Embryonic heart rate, measured on days 13 and 19 of incubation, increased in all enrofloxacin-treated groups and medium and high dose groups of marbofloxacin-treated eggs. Blood biochemistry of chicks sampled at hatch from medium dose groups showed hypoproteinaemia, decreased uric acid and increased triglycerides. Chicks from the enrofloxacin-treated group displayed mild hyperglycaemia and a two-fold rise in the blood urea nitrogen to uric acid ratio. Principal components analysis based on blood biochemistry clearly separated the control bird cluster from both enrofloxacin- and marbofloxacin-treated birds.

**Conclusions:**

Fluoroquinolones induce complex adverse effects on avian embryonic development, considerably reducing the performance of incubated eggs and hatching chicks. Cardiotoxicity, which quickens embryonic heart rate, meant that the total number of heart beats required for embryogenesis was achieved earlier than in the standard incubation period, resulting in pre-term hatching. Our data suggest that enrofloxacin has a higher potential for adverse effects than marbofloxacin. To conclude, care should be taken to prevent exposure of reproducing birds and their eggs to fluoroquinolones.

**Electronic supplementary material:**

The online version of this article (10.1186/s12917-019-1957-y) contains supplementary material, which is available to authorized users.

## Background

Antimicrobials are used to control bacterial diseases of animals and humans worldwide; hence, adverse effects from drug residues and the emergence of resistant bacterial strains may be a serious cause for concern [1–3]. Of the many chemical classes of antibacterial drugs available, fluoroquinolones are amongst the most widely used due to their broad-spectrum bactericidal effects [4–7]. Lipophilic fluoroquinolones, which target a variety of tissues such as the liver and kidney [8], have a long half-life [9] and considerable amounts of fluoroquinolones and their metabolites may reach the environment [3].

It has long been recognised that declining avian populations may act as an indicator of environmental damage associated with pollutants and their adverse effects on reproduction of exposed birds. A wide diversity of pollutants exert effects on several levels, from developing embryos to breeding adult birds [10]. Unlike males, female birds can eliminate toxic contaminants in the contents of their eggs [11–13]; hence, transgenerational exposure may result from bioaccumulation of toxic substances in the female’s body fat or bones and their subsequent mobilisation during egg laying [14]**.** Consequently, bird eggs are commonly used for biological monitoring of environmental levels and distribution of xenobiotics [15–19]. Unfortunately, this excretion route may endanger the avian embryo [13, 20].

It has recently been recognised that fluoroquinolones have detrimental effects on the breeding success of free-ranging avian scavengers [21]. However, further research is necessary to provide evidence-based data regarding the risk of fluoroquinolones for avian embryonic development, e.g. through *in ovo* exposure via egg injection [19, 22]. Using the chicken (*Gallus Gallus domesticus*) as a model avian species, a series of experiments were undertaken to examine toxic effects of the fluoroquinolone antibiotics enrofloxacin and marbofloxacin during egg incubation. Given that both chondrotoxicity and juvenile cartilage growth inhibition have been reported for fluoroquinolones [23], we predict an increase in hatch failure rate and a deterioration in the health status of hatched chicks.

## Results

Fertility within the randomly assigned control and experimental groups varied non-significantly from 84 to 100%. Overall, hatchability was significantly reduced in fluoroquinolone-treated eggs, though not in medium and low dose groups exposed to enrofloxacin and marbofloxacin (Table 1). While the majority of embryonic mortality cases occurred on day 13 of incubation in all groups, single deaths were also observed on day 15 (proportional data for embryonic mortality not shown in Table 1 as they represent the reversed value of hatchability). Total hatching success showed a similar pattern of significant reduction in both low and high exposure groups treated with both enrofloxacin and marbofloxacin (Table 1). Between 31 and 70% of chicks in groups exposed to fluoroquinolones hatched one-day pre-term (i.e. after just 20 days incubation). Aside from medium dosed groups treated with enrofloxacin and marbofloxacin, 15 to 67% of all hatching chicks displayed swollen joint deformities.

**Table 1:** Reproduction parameters achieved with enrofloxacin- and marbofloxacin-treated eggs. Reproduction parameters measured include: Egg numbers set = total eggs of the group incubated, Fertility = percentage of fertilised eggs, Hatchability = percentage of chicks hatching from the fertilised eggs, Pre-term hatching = percentage of chicks hatching on days 19 and 20, Hatching chicks with joint deformity = percentage of chicks showing signs of swollen joints, Total hatching success = percentage of chicks hatching from total eggs set into the incubator. Groups: C1 = untreated control, C2 = sham-treated control eggs (*aqua pro injectione*); E1, E2, E3 = groups exposed to 1, 10 and 100 μg of enrofloxacin per 1 g of egg weight, respectively; M1, M2, M3 = groups exposed to 1, 10 and 100 μg of marbofloxacin per 1 g of egg weight, respectively; ^*^ = *p* < 0.05, ^**^ = *p* < 0.01 (compared against control group C1 or C2)

Groups / Parameters	Untreated control	Sham-treated control	Enrofloxacin-treated Dose			Marbofloxacin-treated Dose		
	C1	C2	Low E1	Medium E2	High E3	Low M1	Medium M2	High M3
Egg numbers set	26	26	20	20	20	20	20	20
Fertility (%)	84	96	90	95	85	95	100	100
Hatchability (%)	90.1	84	55.6 C1^*^C2^*^	84.2	35.3 C1^**^C2^**^	68.4	65 C1^*^	45 C1^**^C2^**^
Pre-term hatching (%)	0	0	70 C1^**^C2^**^	31.3 C1^**^C2^**^	33.3 C1^**^C2^**^	61.5 C1^**^C2^**^	69.2 C1^**^C2^**^	44.4 C1^**^C2^**^
Hatching chicks with joint deformity (%)	0	0	40 C1^**^C2^**^	0	66.67 C1^**^C2^**^	15.38 C1^*^C2^*^	0	55.56 C1^**^C2^**^
Total hatching success (%)	76.9	80.8	50 C2^*^	80	30 C1^**^C2^**^	40 C1^*^C2^**^	65	45 C1^*^C2^*^

**Fig. 1:**
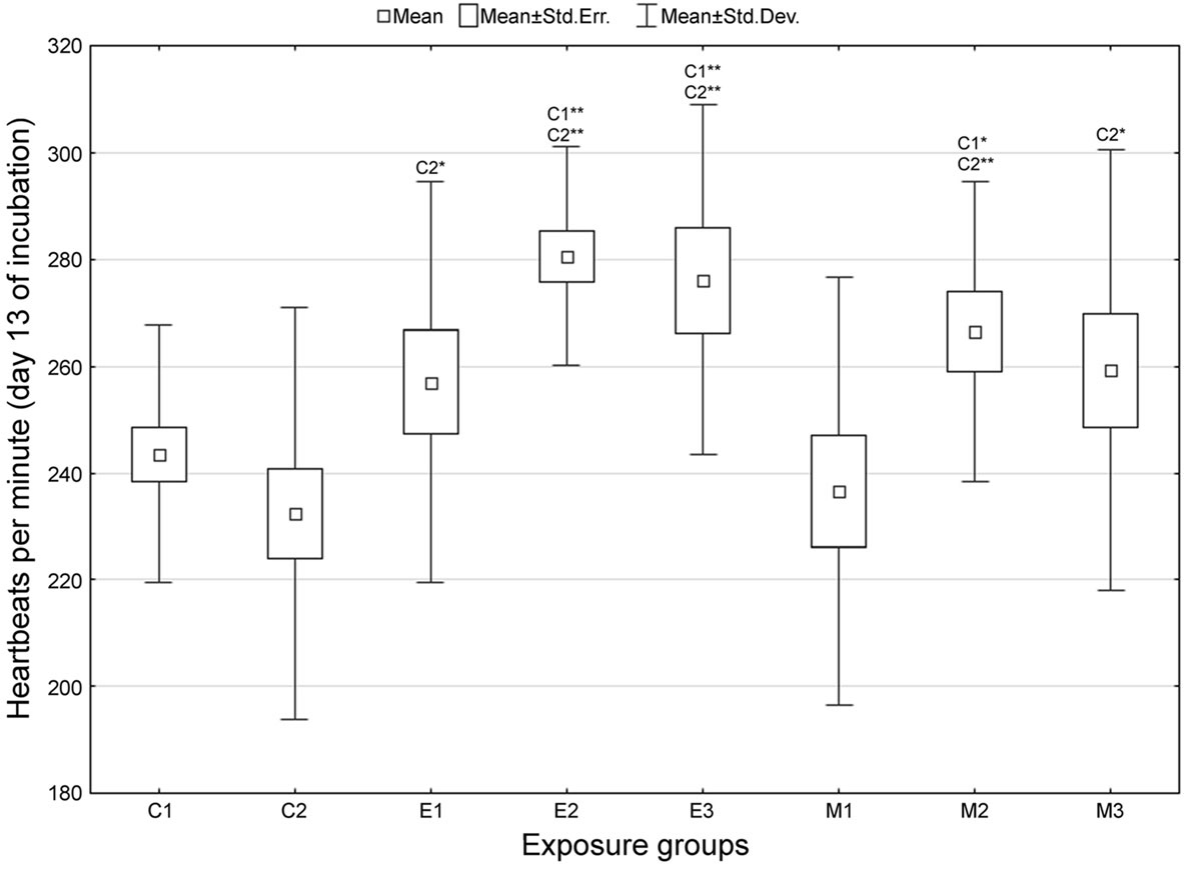
Chicken embryonic heart rate following exposure to fluoroquinolones - day 13 of incubation. Groups: C1 = untreated control, C2 = sham-treated control eggs (*aqua pro injectione*); E1, E2, E3 = groups exposed to 1, 10 and 100 μg of enrofloxacin per 1 g of egg weight, respectively; M1, M2, M3 = groups exposed to 1, 10 and 100 μg of marbofloxacin per 1 g of egg weight, respectively; * = *p* < 0.05, ** = *p* < 0.01 (when compared against control group C1 or C2), n = 26 for each control group and 20 for each exposure group

**Fig. 2:**
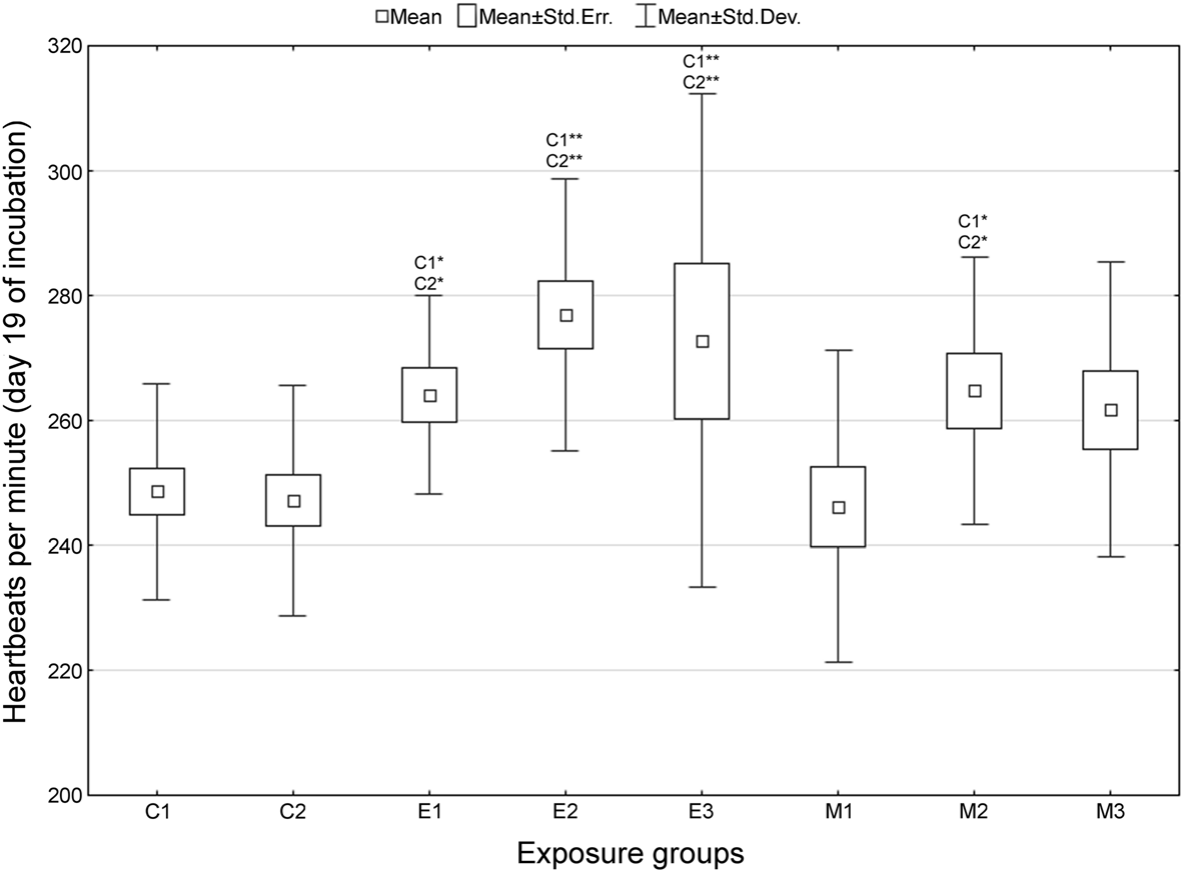
Chicken embryonic heart rate following exposure to fluoroquinolones - day 19 of incubation. Groups: C1 = untreated control, C2 = sham-treated control eggs (*aqua pro injectione*); E1, E2, E3 = groups exposed to 1, 10 and 100 μg of enrofloxacin per 1 g of egg weight, respectively; M1, M2, M3 = groups exposed to 1, 10 and 100 μg of marbofloxacin per 1 g of egg weight, respectively; * = *p* < 0.05, ** = *p* < 0.01 (when compared against control group C1 or C2), *n* = 26 for each control group and 20 for each exposure group

Compared with the untreated and sham-treated control eggs, chicken embryonic heart rate increased in all enrofloxacin-treated groups measured on days 13 and 19 of incubation (Figs. 1 and 2), with the highest increase (20%) being in the medium dose group. Medium and high dose groups of marbofloxacin-treated embryos displayed an increased heart rate on day 13 of incubation (Fig. 1) and on day 19 in the medium dose group (Fig. 2).

**Fig. 3:**
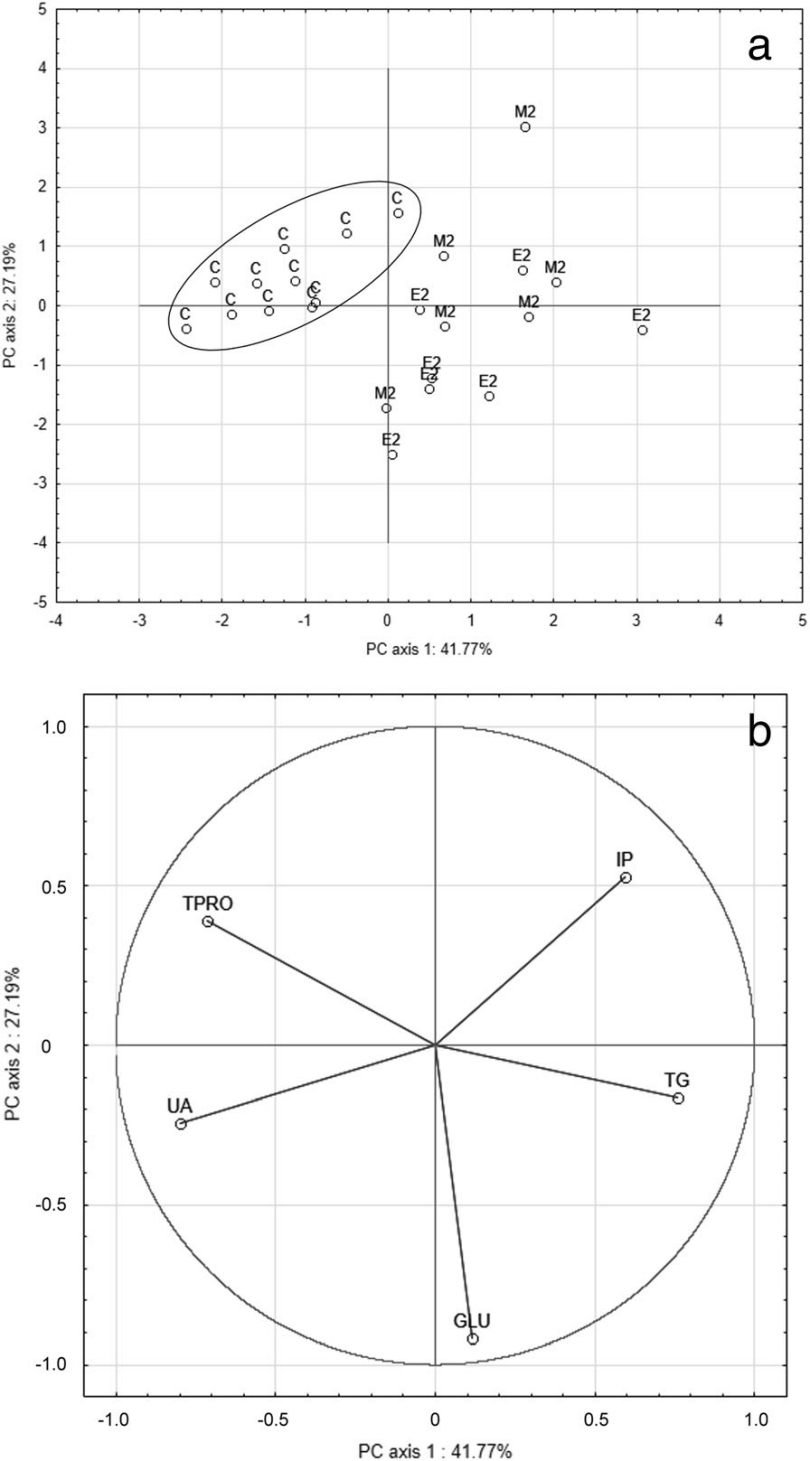
Component score (**a**) and component weight (**b**) plots from principal component analysis of selected blood biochemistry parameters**.** Chick blood samples were obtained on the day of hatching. The cluster of control birds is well separated from all fluoroquinolone-treated birds and the first two components combined explain 68.96% of variation observed. Group labels: C = control birds (data for chicks hatching from untreated and sham-treated control eggs grouped); E2, M2 = birds exposed to fluoroquinolone enrofloxacin (E2 = 10 μg of enrofloxacin per 1 g of egg weight) and marbofloxacin (M2 = 10 μg of marbofloxacin per 1 g of egg weight). Abbreviations: TPRO = total protein, GLU = glucose, UA = uric acid, TG = triglycerides, IP = inorganic phosphorus

Significant hypoproteinaemia, a decrease in uric acid levels and an increase in triglycerides were recorded in chicks from fluoroquinolone-treated eggs (Table 2). The enrofloxacin-treated group was also characterised by mild hyperglycaemia and a ca. two-fold rise in the ratio of blood urea nitrogen to uric acid, while marbofloxacin-treated chicks appeared hyperphosphataemic. Principal components analysis of selected blood biochemistry parameters clearly separated control chicks in a separate cluster from both the enrofloxacin- and marbofloxacin-treated chicks, indicating a marked overall pattern in responses to *in ovo* fluoroquinolone exposure (Fig. 3a). The separation between the control and treatment groups was driven by approximately equal weighting of all variables in the first two principal components (Fig. 3b).

**Table 2:** Blood biochemistry parameters of birds hatching from enrofloxacin- and marbofloxacin-exposed eggs. Values represent mean ± SD; *n* = 11, 7 and 6 in control, enrofloxacin- and marbofloxacin-exposed birds, respectively; ^*^ = *p* < 0.05, ^**^ = *p* < 0.01 (when compared against the control group; as there was no significant difference between the control groups, C1 = untreated control and C2 = sham-treated control eggs (*aqua pro injectione*) were grouped. Blood samples were obtained from chicks on the day of hatching

Parameters	Groups of *in ovo* exposed birds		
	Control	Enrofloxacin-treated Dose E2	Marbofloxacin-treated Dose M2
TPRO (g/l)	24.00 ± 2.45	17.71 ± 1.70^**^	20.00 ± 1.55^**^
BUN (mmol/l)	3.35 ± 1.59	4.46 ± 2.05	3.1 ± 1.14
UA (μmol/l)	429.55 ± 107.33	288.57 ± 139.73^*^	267.00 ± 75.82^*^
BUN/UA	7.99 ± 3.77	17.57 ± 8.91^*^	12.67 ± 5.85
Ca (mmol/l)	2.33 ± 0.41	2.51 ± 0.50	2.52 ± 0.38
IP (mmol/l)	0.74 ± 0.23	0.87 ± 0.16	1.17 ± 0.47^*^
Mg (mmol/l)	0.75 ± 0.05	0.78 ± 0.08	0.75 ± 0.14
GLU (mmol/l)	11.66 ± 0.59	12.76 ± 0.77^*^	12.08 ± 1.11
TG (mmol/l)	0.34 ± 0.09	0.61 ± 0.34^*^	0.61 ± 0.24^*^
TBIL (μmol/l)	5.09 ± 2.43	6.57 ± 1.40	6.00 ± 1.10
T-Chol (mmol/l)	10.13 ± 0.94	9.50 ± 0.55	9.86 ± 1.07
HDL-Chol (mmol/l)	5.99 ± 0.75	5.66 ± 1.06	6.10 ± 1.24
AMY (μkat/l)	11.98 ± 5.42	9.70 ± 2.28	8.33 ± 4.32
AST (μkat/l)	3.39 ± 1.61	4.17 ± 1.97	4.15 ± 1.15
ALP (μkat/l)	69.10 ± 17.96	66.00 ± 14.93	62.52 ± 28.39
CK (μkat/l)	26.29 ± 16.54	37.95 ± 9.99	29.75 ± 10.60
LDH (μkat/l)	27.42 ± 15.04	34.50 ± 17.46	45.95 ± 19.69

Group labels: control birds (C), data for chicks hatching from untreated and sham-treated control eggs were grouped together in this analysis; birds exposed to fluoroquinolone enrofloxacin (dose E2 = 10 μg of enrofloxacin per 1 g of egg weight) and marbofloxacin (dose M2 = 10 μg of marbofloxacin per 1 g of egg weight)

*Abbreviations*: *TPRO* total protein, *BUN* blood urea nitrogen, *UA* uric acid, *BUN/UA* ratio blood urea nitrogen/uric acid, *Ca* calcium, *IP* inorganic phosporus, *Mg* magnesium, *GLU* glucose, *TG* triglycerides, *TBIL* total bilirubin, *T-Chol* total cholesterol, *HDL-Chol* high-density lipoprotein cholesterol, *AMY* amylase, *AST* aspartate aminotransferase, *ALP* alkaline phosphatase, *CK* creatine kinase, *LDH* lactate dehydrogenase

## Discussion

Using the egg injection technique and a standard precocial species model, we show complex adverse effects of fluoroquinolones on avian embryonic development that considerably reduce the performance of incubating eggs. Overall, fluoroquinolone toxicity resulted in decreased hatchability and total hatching success, the spectrum of adverse effects including embryonic mortality (mostly on day 13 of incubation), pre-term hatching, joint deformities in hatched chicks and increased heart rate and biochemical signs of stress.

Although well tolerated in human patients, fluoroquinolones have been reported as prolonging the QT interval, predisposing the heart to life-threatening arrhythmia Torsades de pointes, associated with ventricular tachycardia [24]. In the present study, we also documented cardiotoxic effects of fluoroquinolones on birds, manifested as increased embryonic heart rate (Figs. 1 and 2). Under optimum conditions, species-specific lengths of incubation in egg laying vertebrates are determined by the fixed total number of heartbeats required for development [25]. Depending on the developmental mode of the bird in question (altricial versus precocial), embryonic heart rate also scales allometrically with egg mass [26]. Interestingly, up to two thirds of chicks hatched one-day pre-term in our study, suggesting that the total number of heartbeats required for embryogenesis was achieved earlier than the standard incubation period due to fluoroquinolone-associated cardiotoxicity.

Blood biochemistry of chicks sampled at hatch revealed hypoproteinaemia, which combined with hyperglycaemia and hypertriglyceridemia, is suggestive of prolonged stress [27, 28]. As birds excrete nitrogenous wastes primarily via uric acid, decreased uric acid levels correspond with the findings of hypoproteinaemia. A two-fold rise in the blood urea nitrogen to uric acid ratio in enrofloxacin-treated chicks is also likely to be an indicator of reduced urinary fluid flow [29], while hyperphosphataemia in marbofloxacin-treated chicks may be a renal response. Principal components analysis showed that, in general, the biochemical responses of both enrofloxacin- and marbofloxacin-treated chicks differed from that of the controls.

Fluoroquinolones are important antimicrobials in avian medicine. Though regulated in the poultry industry to exclude residues from poultry products [30], direct medication of poultry and captive birds is sometimes necessary. Enrofloxacin, for example, is effective against egg transmission of *Mycoplasma gallisepticum* [31]; with hatching eggs being treated by dipping in antibiotic solution or through individual egg injection to salvage contaminated embryos. The effectiveness of such antimicrobials, however, can turn from Dr. Jekyll to Mr. Hyde. In wild birds, for example, environmental pollution has become a threat to top predators and/or scavengers due to bioaccumulation and transfer along the food chain [32, 33]. To date, fluoroquinolone antibiotic residues have been identified in the Eurasian griffon vulture *Gyps fulvus* [34], the cinereous vulture *Aegypius monachus* and Egyptian vulture *Neophron percnopterus* [35] and the golden eagle *Aquila chrysaetos* [33]. Such broad-spectrum antimicrobials alter the normal microbiome of birds, with adverse health consequences. Exposure of nestlings to fluoroquinolones is associated with high occurrence and infection intensity of opportunistic fungal pathogens, as previously reported for vultures with oral candida-like lesions [35], and may also attenuate immune responses [36]. Aside from both medium dosed groups, we observed swollen joints with deformities in enrofloxacin- and marbofloxacin-treated groups, most likely due to the chondrotoxic effect of fluoroquinolones [23]. This biphasic dose-response is suggestive of hormesis mechanisms [37] operating in adverse effects of fluoroquinolones on avian embryonic development. Proper functioning of the locomotor apparatus is considered essential for movement of the chick during hatching; nevertheless, our own study showed that viable chicks with affected joints were able to hatch.

Avian embryos represent a unique model for studying reproductive toxicity due to the ease with which they can be manipulated experimentally [38]. Compared with altricial modes of development, precocial chicks are more developed when they hatch, meaning that critical periods for essential physiological systems to achieve functional competency are completed during incubation. In precocial birds exposed to toxic substances, therefore, higher sensitivity to adverse effects should occur during incubation. At this time, however, we lack altricial toxicological models to test this hypothesis [38].

## Conclusions

Higher toxicity awareness among practicing veterinarians may reduce adverse reproductive effects of fluoroquinolones in birds. Importantly, our data suggest that enrofloxacin has a higher potential for adverse effects than marbofloxacin.

## Methods

### Experimental design

To evaluate the effect of fluoroquinolones on avian embryonic development, chicken eggs from a single batch were randomly divided into experimental groups. Eggs were injected with three fluoroquinolone doses of 1, 10 and 100 μg/g of egg weight on day 0 (i.e. prior to incubation). The selected dosing regime was environmentally relevant. Medium doses are of the same order of magnitude with concentrations of fluoroquinolones determined in eggs of wild birds of prey [21]. Likewise, these general doses would be selected in the treatment to salvage eggs with bacterial infections. In addition to untreated controls (C1), sham-treated controls (C2) were prepared by injecting with an adjusting solution (*aqua pro injectione*). A total of 20 eggs were used on each yolk-injection treatment group and 26 eggs for both controls. Each egg was numbered using a pencil and then set in an incubator with temperature and humidity control and an automatic turning device. Incubation was initiated after 4 days of egg storage and fertility and embryo viability checked by candling the eggs on day eight of incubation. All eggs containing live embryos were placed back into the incubator, while those that appeared not to contain a live embryo were opened and examined under a dissecting microscope to distinguish between infertility and early embryonic death, each egg then being recorded as fertile, infertile, viable or unviable due to embryonic death. On days 13 and 19 of incubation, a non-invasive monitoring device (Buddy Egg, Avitronics, United Kingdom) was used to check for embryo viability and to measure embryo heart rate. Dead embryos from day 15 were recorded as late embryonic death. Other data collected during the study included gross embryo abnormalities, length of incubation period from setting to hatching and reproductive indices such as fertility (percentage of fertile eggs), hatchability (percentage of chicks hatching from fertile eggs) and total hatching success (percentage of chicks hatching from all eggs set for incubation) [39].

### Experimental substances

Two commercially available veterinary injection solutions were used for the experiment, i.e. 100 mg/ml Enroxil (Krka, Slovenia) and 100 mg/ml Marbocyl (Vétoquinol, France), containing enrofloxacin and marbofloxacin, respectively. Three fluoroquinolone exposure doses of 1, 10 and 100 μg/1 g of egg weight were adjusted using *aqua pro injectione* such that a total volume of 100 μl was injected into each egg. The effect of propylene glycol used as a solvent and a preservative in veterinary products was considered negligible due to its trace quantity combined with extremely low toxicity in the egg-injected bolus.

### Egg injection technique and incubation

Hubbard broiler chicken eggs were purchased as a batch from a commercial supplier (DENAS poultry breeder, Studenka, Czech Republic). The experimental substances were injected into the egg yolks on day 0 (prior to incubation) as described elsewhere [22]. Briefly, the egg surface over the air cell was disinfected with 70% ethanol, a hole drilled through the eggshell and the yolk injected with the experimental solution. The eggshell was then sealed with tissue glue (Surgibond, SMI AG, St. Vith, Belgium) and paraffin wax and set for incubation. Eggs were incubated in a Favorit S + H 288 TOP Digital incubator (HEKA-Brutgeraete, Rietberg, Germany) using standard conditions (temperature 37.5–37.6 °C; humidity 60%, increased to 65% for the final 3 days of incubation) and automatic turning.

### Biochemistry

Blood (0.5 ml) was collected from chicks at hatch by cardiac puncture using a heparinised Omnican 0.30x12mm insulin set (Braun, Germany), and processed as previously described [27, 40, 41]. The chicks were anaesthetised using isoflurane for the procedure of blood sampling and sacrificed by decapitation afterwards. Whole blood samples were analysed immediately using an automated analyser (SPOTCHEM™ EZ SP-4430, ARKRAY, Japan) for total protein (g/l), blood urea nitrogen (mmol/l), uric acid (mmol/l), ratio of blood urea nitrogen to uric acid, calcium (mmol/l), inorganic phosphorus (mmol/l), magnesium (mmol/l), glucose (mmol/l), triglycerides (mmol/l), total bilirubin (μmol/l), total cholesterol (mmol/l), high-density lipoprotein cholesterol (mmol/l), amylase (μkat/l), aspartate aminotransferase (μkat/l), alkaline phosphatase (μkat/l), creatine kinase (μkat/l) and lactate dehydrogenase (μkat/l). A sufficient sample size for blood biochemistry comparison was only obtained from medium dose groups treated with enrofloxacin and marbofloxacin. As there was no significant difference between control groups C1 and C2, the biochemical data for the controls was grouped.

### Statistical analysis

Additional file 1: Table S1 provides data generated and analysed during this study. Statistica for Windows® 10 (StatSoft, Inc., Tulsa, OK, USA) was used to compare different experimental groups against both controls using the following tests: one-way analysis of variance (ANOVA), post-hoc analysis of means by the LSD test, Levene’s method for testing homogeneity of variance, log-transformation of non-homogenous parameters prior to analysis and the non-parametric Kruskal-Wallis test. Reproductive parameters were compared by testing the difference between two proportions. Multivariate analysis of blood biochemistry parameters (total protein, glucose, uric acid, triglycerides and inorganic phosphorus) was performed using principal components analysis. Levels of significance were either *p* < 0.05 or *p* < 0.01.

## Additional file

Additional file 1: **Table S1.** Reproductive toxicity of fluoroquinolones in birds: supporting data. Measured data are given for 1. Chicken embryonic heart rate following exposure to fluoroquinolones (heartbeats per minute) and 2. Blood biochemistry parameters of birds hatching from enrofloxacin- and marbofloxacin-exposed eggs. (XLSX 19 kb)

## Data Availability

All supporting data accompany this published article as Additional file 1: Table S1.

## Abbreviations

ALP: Alkaline phosphatase
AMY: Amylase
ANOVA: Analysis of variance
AST: Aspartate aminotransferase
BUN: Blood urea nitrogen
BUN/UA: Ratio blood urea nitrogen/uric acid
Ca: Calcium
CK: Creatine kinase
GLU: Glucose
HDL-Chol: High-density lipoprotein cholesterol
IP: Inorganic phosporus
LDH: Lactate dehydrogenase
LSD test: Least significant test
Mg: Magnesium
TBIL: Total bilirubin
T-Chol: Total cholesterol
TG: Triglycerides
TPRO: Total protein
UA: Uric acid
